# Physical Properties of an Efficient MAPbBr_3_/GaAs Hybrid Heterostructure for Visible/Near-Infrared Detectors

**DOI:** 10.3390/nano14181472

**Published:** 2024-09-10

**Authors:** Tarek Hidouri, Maura Pavesi, Marco Vaccari, Antonella Parisini, Nabila Jarmouni, Luigi Cristofolini, Roberto Fornari

**Affiliations:** 1 Department of Mathematical, Physical and Computer Sciences, University of Parma, Parco Area delle Scienze 7/A, 43124 Parma, Italy; maura.pavesi@unipr.it (M.P.); marco.vaccari@unipr.it (M.V.); antonella.parisini@unipr.it (A.P.); luigi.cristofolini@unipr.it (L.C.); roberto.fornari1@unipr.it (R.F.); 2 Instituto Italiano di Tecnologia, Via Morego 30, 16163 Genova, Italy; nabila.jarmouni@iit.it; 3 Institute of Materials for Electronics and Magnetism, National Research Council (CNR), Parco Area delle Scienze 37/A, 43124 Parma, Italy

**Keywords:** hybrid materials, GaAs, perovskites, MAPbBr_3_, photodetection, integration, carrier localization

## Abstract

Semiconductor photodetectors can work only in specific material-dependent light wavelength ranges, connected with the bandgaps and absorption capabilities of the utilized semiconductors. This limitation has driven the development of hybrid devices that exceed the capabilities of individual materials. In this study, for the first time, a hybrid heterojunction photodetector based on methylammonium lead bromide (MAPbBr_3_) polycrystalline film deposited on gallium arsenide (GaAs) was presented, along with comprehensive morphological, structural, optical, and photoelectrical investigations. The MAPbBr_3_/GaAs heterojunction photodetector exhibited wide spectral responsivity, from 540 to 900 nm. The fabrication steps of the prototype device, including a new preparation recipe for the MAPbBr_3_ solution and spinning, will be disclosed and discussed. It will be shown that extending the soaking time and refining the precursor solution’s stoichiometry may enhance surface coverage, adhesion to the GaAs, and film uniformity, as well as provide a new way to integrate MAPbBr_3_ on GaAs. Compared to the pristine MAPbBr_3_, the enhanced structural purity of the perovskite on GaAs was confirmed by X-ray Diffraction (XRD) upon optimization compared to the conventional glass substrate. Scanning Electron Microscopy (SEM) revealed the formation of microcube-like structures on the top of an otherwise continuous MAPbBr_3_ polycrystalline film, with increased grain size and reduced grain boundary effects pointed by Energy-Dispersive Spectroscopy (EDS) and cathodoluminescence (CL). Enhanced absorption was demonstrated in the visible range and broadened photoluminescence (PL) emission at room temperature, with traces of reduction in the orthorhombic tilting revealed by temperature-dependent PL. A reduced average carrier lifetime was reduced to 13.8 ns, revealed by time-resolved PL (TRPL). The dark current was typically around 8.8 × 10^−8^ A. Broad photoresponsivity between 540 and 875 nm reached a maximum of 3 mA/W and 16 mA/W, corresponding to a detectivity of 6 × 10^10^ and 1 × 10^11^ Jones at −1 V and 50 V, respectively. In case of on/off measurements, the rise and fall times were 0.40 s and 0.61 s or 0.62 s and 0.89 s for illumination, with 500 nm or 875 nm photons, respectively. A long-term stability test at room temperature in air confirmed the optical and structural stability of the proposed hybrid structure. This work provides insights into the physical mechanisms of new hybrid junctions for high-performance photodetectors.

## 1. Introduction

Lead halide perovskites with an APbX_3_ stoichiometry (A is methylammonium, formamidinium, or cesium; Pb is lead; and X is I^−^, Br^−^, or Cl^−^) have re-emerged as an exciting class of semiconductors for optoelectronic applications, owing to their excellent optical and electrical properties, such as their large absorption coefficients, long carrier lifetimes, and balanced hole and electron transport [1,2,3,4,5,6].

They have been utilized in high-performance solar cells [7], light-emitting diodes (LEDs) [8], lasers [9], and ultraviolet-to-infrared photodetectors [10], as well as X- and γ-ray detectors [11].

From a scientific point of view, perovskite single crystals are needed to study the basic properties of the material, while, from a commercial point of view, single-crystal perovskite devices are scarcely used because of their sensitivity to oxygen, moisture, light, and thermal conditions [12].

Thus, integrating perovskite layers into other conventional materials is a new way of achieving high device efficiency levels using cost-effective deposition methods.

For perovskite deposition, several techniques such as spin coating, inkjet printing, mechanical slicing, space-confined crystallization, chemical vapor deposition, electrodeposition, etc. were previously reported [13,14,15]. Such epitaxial techniques lead to uniform films but are expensive and time consuming, and they require the development of specific technologies. On the other hand, spin coating has proven to be a simple way of coating a surface with a uniform film by controlling the speed, acceleration, and spinning time, which is easy from a technical point of view [16]. Due to the low-cost and easy fabrication of organic–inorganic hybrid perovskite materials via spin coating, researchers were stimulated to combine perovskites with other material groups to obtain hybrid heterojunctions for optoelectronic devices; for example, perovskites have been grown on common substrates such as Si, SnO_2_, GaN, quartz, gold, Ga_2_O_3_, and sapphire, etc. [17,18,19]. That integration provided optoelectronic devices that combined the advantages of perovskites with those of IV or III–V semiconductors, as well as with those of other inorganic hetero-substrates. While lead halide perovskite performs well in terms of photodetection, a common problem for practically all perovskite materials and devices is its poor adhesion to the substrate surface and its instability with respect to ambient temperature, humidity, and illumination. This strongly limits the industrial utilization of perovskite for optoelectronic devices. In contrast to this present work, so far, most reported perovskite devices are manufactured in glove boxes with nitrogen protection, which causes them to lose their performance claims when exposed to actual environmental conditions. It must also be noted that initial research on perovskite photodetectors was essentially focused on single crystals and deposition on ITO/glass substrates, with little attention paid to thin-film research. Liyun Zhao and his coworkers have developed a heteroepitaxial, highly oriented cesium lead bromide (CsPbBr_3_) single crystal on c-wurtzite GaN/sapphire templates using chemical vapor deposition [15,20,21]. The proposed heterojunction, however, needs optimization, concerning, for example, the poor surface covering that affects its physical properties and its overall cost, which hinders commercialization. Jingli Ma et al. [22] studied Cs_3_Cu_2_I_5_ perovskite on Ga_2_O_3_ templates via dual-source vapor co-deposition. The film showed good optoelectronic properties, but it only responded to photons with energy in a narrow window. Linpeng Dong et al. [19] fabricated MAPbI_3_/β-Ga_2_O_3_ photodetectors with good performances, but these were affected by instability, typical of iodide-based perovskites. Generally, different approaches have been thus investigated to increase their stability and emission efficiency, such as the post-annealing method [23], the two-step fabrication method [24], and protection by passivation [25,26]. However, to the best of our knowledge, no reports have been provided on the deposition of lead halide perovskites on GaAs.

The subject of this present work is the preparation of MAPbBr_3_/GaAs heterojunctions for the first time and their relevant characterizations. MAPbBr_3_ exhibits high open-circuit voltage due to its large bandgap. Furthermore, its photoluminescence emission wavelength can be tuned by changing the halide element concentration, and it offers good stability against humidity. This makes MAPbBr_3_ suitable for optoelectronics such as wavelength tunable light-emitting diode devices and photodetectors in combination with suitable semiconductors. GaAs presents several advantages over silicon, including higher electron mobility, hole mobility similar to that of silicon, moisture resistance, and a direct bandgap, which makes it a suitable candidate for advanced near-infrared (NIR) light detection systems.

Considering the lack of systematic research on the preparation and characterization of lead halide perovskites on gallium arsenide, we concentrated on the fabrication of a novel heterojunction photodetector based on MAPbBr_3_ films deposited on GaAs. We demonstrate the easy preparation of MAPbBr_3_ perovskite films by optimizing the soaking time of the antisolvent. The film is made of interconnected micron-sized crystals on (001) n-GaAs. These crystals are bigger than those obtained in pristine MAPbBr_3_. The proposed method provides a simple and viable route for the preparation of APbX_3_/GaAs heterojunctions. Their structural, morphological, and photoelectrical properties were correlated with the deposition parameters and optimized to assess the potential of the hybrid MAPbBr_3_/GaAs heterojunction in high-performance optoelectronic devices. Specifically, the heterojunction was designed for photodetection and prepared in harsh conditions (in air, no doping, no carrier transporting layers, and no encapsulation) exploiting the background p-type characteristic of MAPbBr_3_ [2,5] and a commercialized n-GaAs.

No direct deposition of MAPbBr_3_ on GaAs, in air without encapsulation, and carrier transport layers were reported so far, even though this approach offers a direct, easy solution towards perovskite integration with GaAs. This is a distinctive feature of this present work.

## 2. Experimental Details

### 2.1. Preparation of MAPbBr_3_/GaAs Heterojunction

(i) Substrates and cleaning: Commercial GaAs wafers from Biotain Crystal Co., Ltd., company (Longyan, China) grown by vertical gradient freezing with n-type conductivity and carrier concentration, n ≈ 3–4 × 10^17^ cm^−3^, were used as the substrate for the MAPbBr_3_ perovskite. The GaAs wafers (up to 1 cm^2^) were cleaned and hydrophilic-treated. The glass substrate was cleaned with detergent and sequential ultrasonic treatment in acetone for 15 min and isopropyl alcohol for 15 min.

(ii) Solvents and precursors: MABr, PbBr_2_, and dimethyl sulfoxide (DMSO) were dissolved in dimethylformamide (DMF anhydrous, 99.8%). The molar ratio, DMSO:PbBr_2_, was fixed at (2:1) the MABr:PbBr_2_ ratio was optimized to be (3:1). All the reagents were used as received without further purification.

(iii) Preparation of precursor solution: 1 mmol of PbBr_2_ (99.999%, Merk Life Science S.r.l., Milan, Italy,), 3 mmol of MABr (336 mg), and 2 mmol of DMSO (156 mg > 99.9%, Merk Life Science S.r.l., Milan, Italy) were dissolved in 494 mg of DMF (99.8%, Merk Life Science S.r.l., Milan, Italy) under continuous stirring to ensure dissolution. The transparent solution was fully dissolved at room temperature (RT) and was filtered using a 0.45 μm pore-sized syringe filter before use. The perovskite layer was obtained by spreading the xMABr·PbBr_2_·2DMSO solution on the target substrate.

(iv) Deposition on GaAs: 150 µL of the solution was dropped on GaAs. First, it was spun at 800 rpm for 20 s (without antisolvent), leading the precursors to spread on the surface as quasi-separated droplets. Then, the spinning continued at a stable acceleration of 1000 rpm/s up to a final speed of 1500 rpm. Changing the speed and/or acceleration at this limit has no effect on the morphology of the sample. However, one must keep the same strategy—low speed at first stage and high speed at the final stage. A total quantity of 0.5 mL (550 mg) of diethyl-ether was added by sequential dropping during spinning to achieve higher reactivity and better cover the surface.

The time of the sequential dropping of the antisolvent was experimentally adjusted in order to maximize the surface covering.

**Figure 1:**
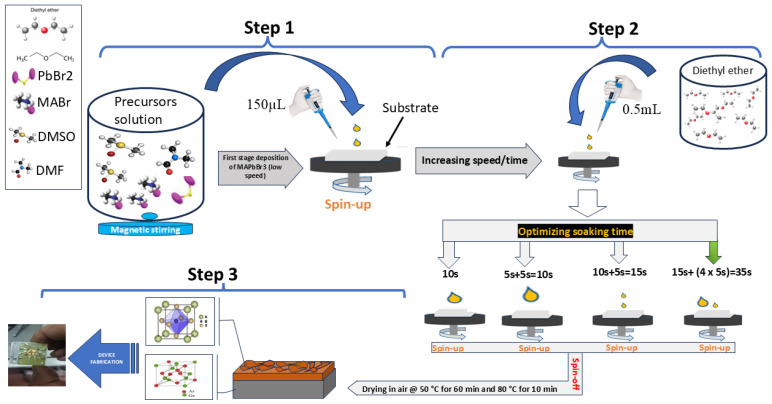
Schematic presentation of the multistep process for the deposition of MAPbBr_3_ on GaAs. Acronyms of the chemical compounds are provided in the article text.

Finally, the spin-coated films were heated slowly at 50 °C for 60 min, then at 80 °C for 10 min in air to evaporate solvents. Figure 1 shows a schematic picture of the multistep deposition procedure. Table 1 reports the deposition process as a function of the soaking time of the antisolvent and the list of the deposited samples. The best results were obtained after a longer soaking time (sample S4 in Table 1), corresponding to the process marked by the green arrow in Figure 1. An explication of the soaking time sequencing can be found in Supporting Information, Note S1.

### 2.2. Characterization

UV–VIS absorption spectra were recorded with a Cary 300 spectrophotometer (Shirley, NY, USA). PL measurements were carried out using Continuous Wave (CW) excitation at 500 nm by an Ar ion laser (Spectra-Physics, Stahnsdorf, Germany), spot size ∼0.007 cm^2^, under variable light excitation power and temperature conditions (10 K–300 K). During temperature-dependent PL measurements, samples were kept in a closed-cycle helium cryostat. The PL emission, dispersed by a high-resolution spectrometer (Jobin-Yvon monochromator (Spectra-Physics, Stahnsdorf, Germany): focal length 0.6, resolution: 10 Å/mm width of the input slot, two 600 line/mm diffraction gratings), was then detected by a phototube with a built-in amplifier (up to ∼10^4^). The PL lifetime was measured by a Perkin-Elmer LS-50B luminescence spectrometer (pulsed laser at 505 nm, NanoLED-405L (Spectra-Physics, Stahnsdorf, Germany)), using pulses of 3 ns duration with a repetition rate of 10 Hz at a stable and/or varied photon density rate ranging from 1 × 10^16^ cm^−3^ to 15 × 10^16^ cm^−3^.

XRD measurements were performed with a PANalytical Empyrean X-ray diffractometer (Madison, WI, USA) equipped with a 1.8 kW Cu Kα ceramic X-ray tube and a PIXcel3D 2 × 2 area detector. This works at 45 kV and 40 mA under ambient conditions with a parallel beam geometry and symmetric reflection mode. The final sample was put onto a zero-diffraction silicon substrate.

SEM measurements were performed using a Field-Emission SUPRA40 (Zeiss, Baden-Wurttemberg, Germany) SEM microscope equipped with a GEMINI FESEM detection column (Zeiss, Baden-Wurttemberg, Germany) featuring an in-lens detector. Energy-Dispersive Spectroscopy (EDS) measurements were performed using a Silicon Drift Detector (SDD) X-act 10 mm^2^ LN2-free (Oxford Instruments, Abingdon, UK) mounted on the same instrument, a SUPRA40 Zeiss SEM microscope. Cathodoluminescence measurements (CL-SEMs) were performed with a panchromatic detector coupled with a lightguide (Zeiss, Baden-Wurttemberg, Germany).

Additionally, 50 nm thick gold (Au) circular electrodes were deposited on the MAPbBr_3_/n-GaAs film by a thermal evaporation process through a mask with a diameter of 0.4 mm. Then, electrodes were wired by using silver conductive paste. The sample was kept at room temperature during metal deposition to avoid any additional annealing effects.

## 3. Results and Discussion

### 3.1. Homogeneity of MAPbBr_3_ Films on GaAs

The successful coverage of a substrate is governed by factors such as the precursor quantity deposited on the substrate. This determines the kinetics of grain growth, grain coalescence, and the subsequent formation of continuous thin film. Non-stochiometric conditions are preferable, as they prevent the formation of undesired phases at the interface that may lead to perovskite decomposition.

**Figure 2:**
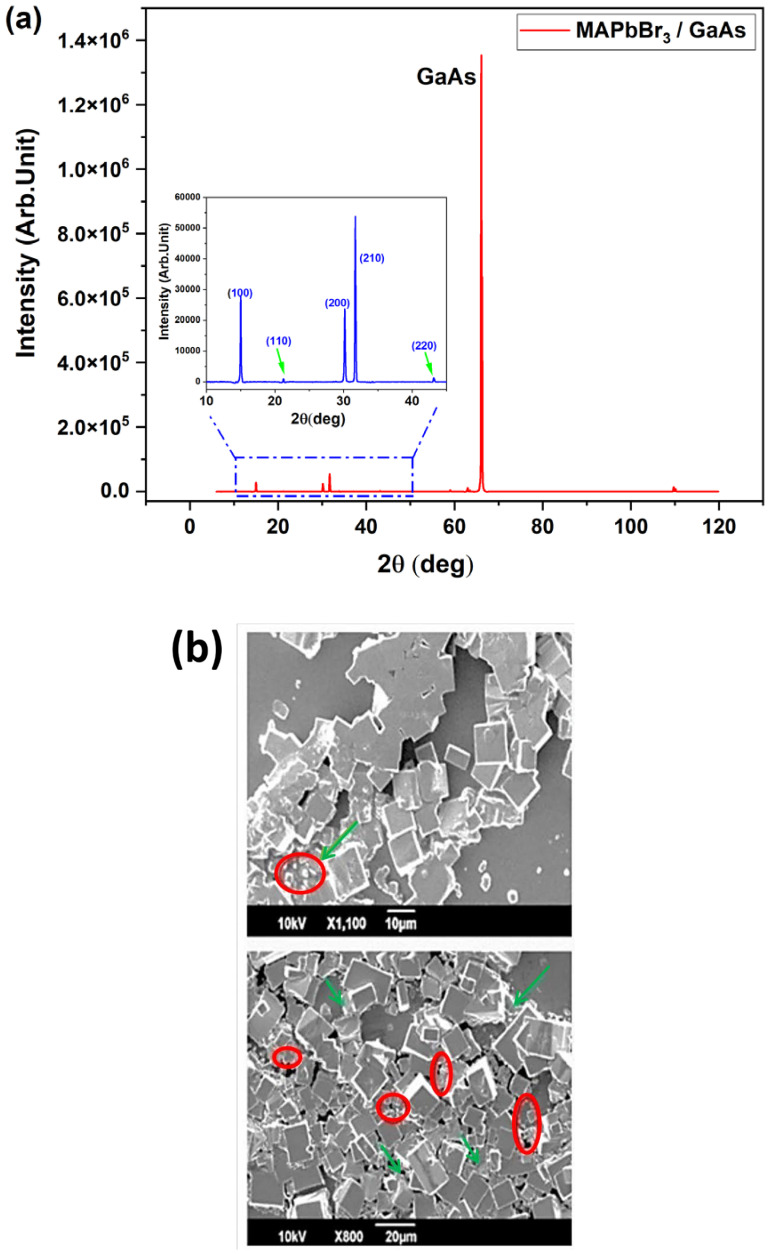
(**a**) XRD spectrum of MAPbBr_3_/GaAs (sample S *, see Table 1) with a zoomed portion of the spectrum to highlight the MAPbBr_3_ peaks; (**b**) SEM images showing poor (upper image) and improved coverage (lower image) by the perovskite microcubes and the formation of an underlying layer (red circles).

Let us start the discussion by analyzing sample S * in Table 1. The MAPbBr_3_ tends to form individual islands on GaAs. The XRD profile in Figure 2a and SEM images in Figure 2b correspond to the crystallization within a single island. The diffraction peaks of the perovskite are located at 15°, 21.25°, 30°, and 45° (inset of Figure 2a) and correspond to the (001), (011), (002), and (003) crystal planes of MAPbBr_3_, respectively [27]. Compared to the XRD profile of the control sample on glass (S ^g^) (Figure S1, Supporting Information), it is clear that the (111), (021), (211), and (022) crystal planes disappeared upon deposition on GaAs. This suggests that the GaAs surface exerts positive action towards the formation of high-quality crystalline films on GaAs.

Nevertheless, the SEM image evidences some coverage issues, with 11 µm sized single or coalesced microcubes randomly distributed over the surface. Such a result is in good agreement with the grain size range reported in Ref. [28]. This size is larger than that in S ^g^, which shows an average grain size of 0.7 µm (Figure S1, Supporting Information). This confirms the validity of the proposed deposition recipe. Note that a larger grain size will mitigate ion migration, which, in turn, can significantly affect the long-term stability of the devices [28]. Furthermore, larger grains can decrease the charge recombination at the grain boundaries, enhancing the carrier transport phenomenon, with clear benefits in terms of photodetection efficiency.

Due to the presence of gallium (Ga) and arsenic (As), the surface reaction between PbBr_2_ and MABr can be delayed. Indeed, the MABr excess acts as a continuous host-layer (green arrows in Figure 2b) and a passivation-like layer or an underlying layer (red circles).

Subsequently, the MAPbBr_3_ microcube takes form within this original layer. The incomplete coverage may probably result from the slow diffusion of the perovskite molecules on the GaAs surface upon spinning, the high crystallization rate, and/or the inhomogeneous distribution of the antisolvent. Note that if the reaction time of the antisolvent with the precursor solution is very short, film thickening becomes dominant over the lateral growth. This gives rise to the formation of isolated droplets on the surface. This Volmer–Weber (V-W)-like growth mode limits total covering [29]. To overcome this limitation, the antisolvent soaking time is adjusted to enhance its diffusion in parallel with the nonstoichiometric conditions. This will increase the nucleation density, slow down the crystallization rate, and improve the coalescence.

**Figure 3:**
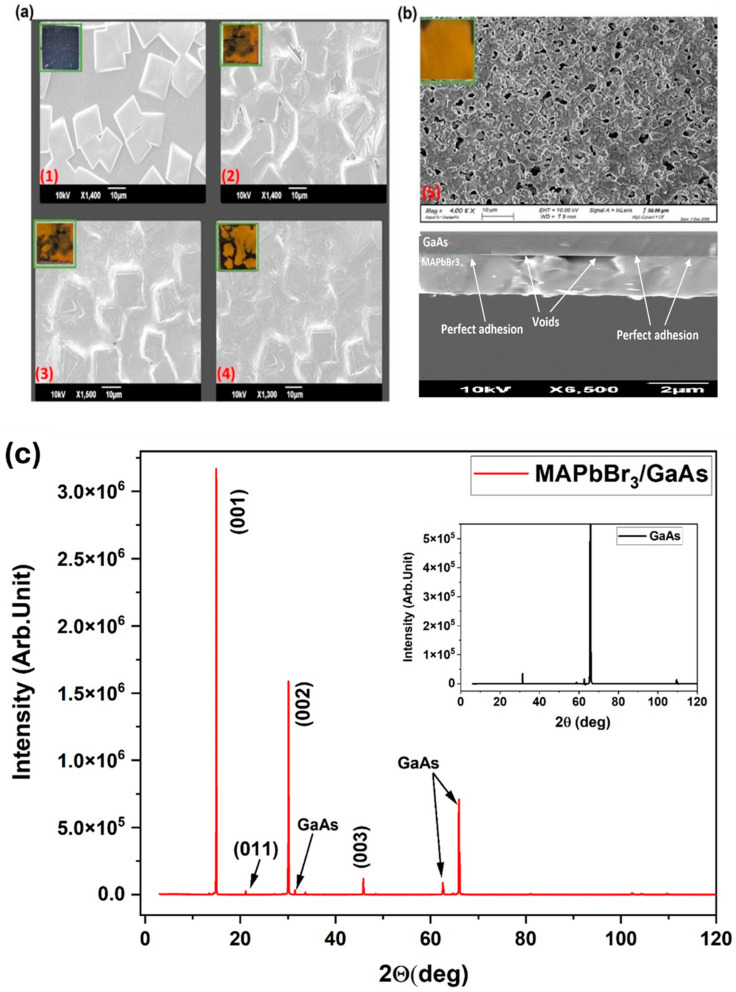
(**a**) Top-view SEM images showing the effect of the antisolvent soaking time on the morphology of MAPbBr_3_/GaAs, with reference to Table 1 (image 1, sample S *); (image 2, sample S1); (image 3, sample S2); (image 4, sample S3); and (image 5, sample S4). The corresponding insets are photos of the sample surfaces. (**b**) Top-view and cross-section SEM of S4 (image 5) with 1 cm^2^ full surface coverage, as seen in the surface photo in the inset; (**c**) XRD diffractogram of S4.

SEM images in Figure 3a show the effect of the proposed preparation procedure. A full continuous MAPbBr_3_ layer covers the GaAs substrate. It is made of closely packed inhomogeneous cuboid-like crystals ((0.7 µm × 1 µm) and (1.2 µm × 1.7 µm)) is distributed on the top of continuous MAPbBr_3_ regions. The presence of pinholes may be due to the size of the substrate, which affects the diffusion rate of the antisolvent and the crystallization process [30,31].

The reached thickness of MAPbBr_3_ on GaAs is ~1.8 µm, compared to 867 nm on the glass substrate (S0 *) (Figure S2, Supporting Information). From Figure 3b, the adhesion on the GaAs surface appears to be limited; perfectly adherent regions are alternate to interface voids. Similar behavior has been reported for CsPbBr_3_ [32]. Parasitic reactions between the precursors and the formation of spurious by-products may be responsible for imperfect film adhesion. The (001) GaAs surface is terminated by 1–10 arsenic (As) dimers [33]. Dimers will slow down the surface reaction time allowed for the mixture between PbBr_2_ and MABr. When heating, the arsenic reacts with bromine to produce arsenic(III)-bromide. This reaction takes place at 50–80 °C (the film annealing temperature) according to the following:

$$2\mathrm{As}+3{Br}_{2}\overset{T(50\;{}^{\circ}C–80\;{}^{\circ}C)}{\to }2As{Br}_{3}$$

On the other hand, arsenic does not react with air at room temperature without the presence of moisture. With moisture, arsenic slowly reacts, forming arsenic(V)-oxide when heated.

$$4\mathrm{As}+5O_{2}\overset{T}{\to }2{As}_{2}O_{5}$$

The non-homogeneous distribution of the precursor solution with the antisolvent may thus cause a lack of adhesion in some regions of the substrate surface. This is more pronounced in cases of imperfect contact between the sample and the annealing hotplate (inhomogeneous heating).

Polycrystallinity is confirmed by the XRD (Figure 3c). The intense diffraction peaks at 15° and 30° of (001) and (002) indicate a highly oriented polycrystal structure and good crystallinity. Two major diffraction peaks of GaAs are visible at 31.75° and 66°. No diffraction peaks that may be ascribed to PbBr_2_ (usually at 18.61°) or MABr (at 10°) were observed. This confirms that PbBr_2_ and MABr are entirely converted to MAPbBr_3_, and no phase separation occurs.

**Figure 4:**
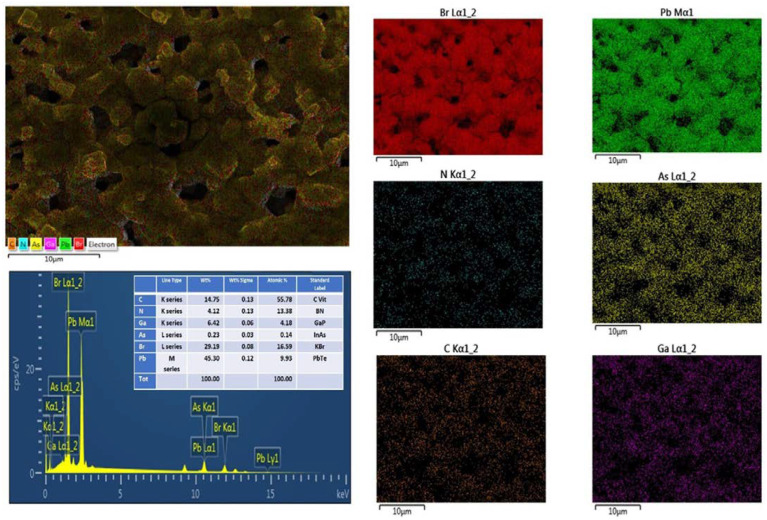
Typical energy-dispersive X-ray spectroscopy (EDX) elemental mapping images showing the EDS layered image of S4 and corresponding element analysis.

To view the element distribution in the optimized sample, EDS mappings (Figure 4) were performed on large (10–100 µm) portions of the film. It can be seen that Br and Pb are uniformly distributed and maintain the stoichiometric ratio. Further discussion on element distribution can be found in Note S2 (Supporting Information). Figure S3 (Supporting Information) shows that the bulk of the layer, under the surface cubes, shows some local composition fluctuations (MABr:PbBr = 1.5:1). This local perturbation affects the PL response by giving rise to potential fluctuation and carrier localization. This point will further be discussed in the PL section.

**Figure 5:**
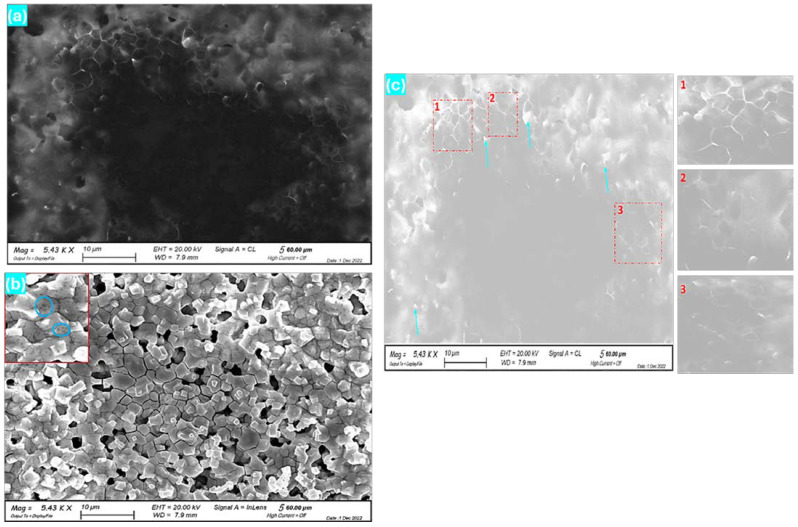
SEM-CL images of the MAPbBr_3_/GaAs heterostructure (sample S4). (**a**) The original CL image. (**b**,**c**) Treated SEM-CL images for better clarity.

The results of SEM-CL measurements performed on the optimized sample, S4, are shown in Figure 5a, with some dark spots indicated by blue circles in the zoomed part of Figure 5b (upper left corner). These are attributed to structural defects inside the grains and/or precipitates. The edges of crystals and grain boundaries are seen to produce a stronger emission. The distribution of grains and their boundaries are, therefore, very well resolved in CL. A few small areas with comparatively higher emissions (brighter spots indicated by blue arrows in Figure 5c) are also observed. This is also confirmed upon observation of zones 1, 2, and 3 in Figure 5c. By comparing the grain boundaries with the grain interiors, one can note that the boundaries are richer in bromine and give a brighter emission [34]. This supports the idea that V_Br_ is a deep center that may play a detrimental role in these materials. Moreover, we must consider that photogenerated carriers encounter difficulties in crossing grain boundaries and hopping between grains to reach the device electrodes. Preventing grain boundaries (i.e., producing large crystallites) is thus important in order to increase device efficiency. Larger crystallites enhance the mobility of photocarriers and reduce their recombination via deep levels or trapping phenomena, which minimize device losses [35].

**Figure 6:**
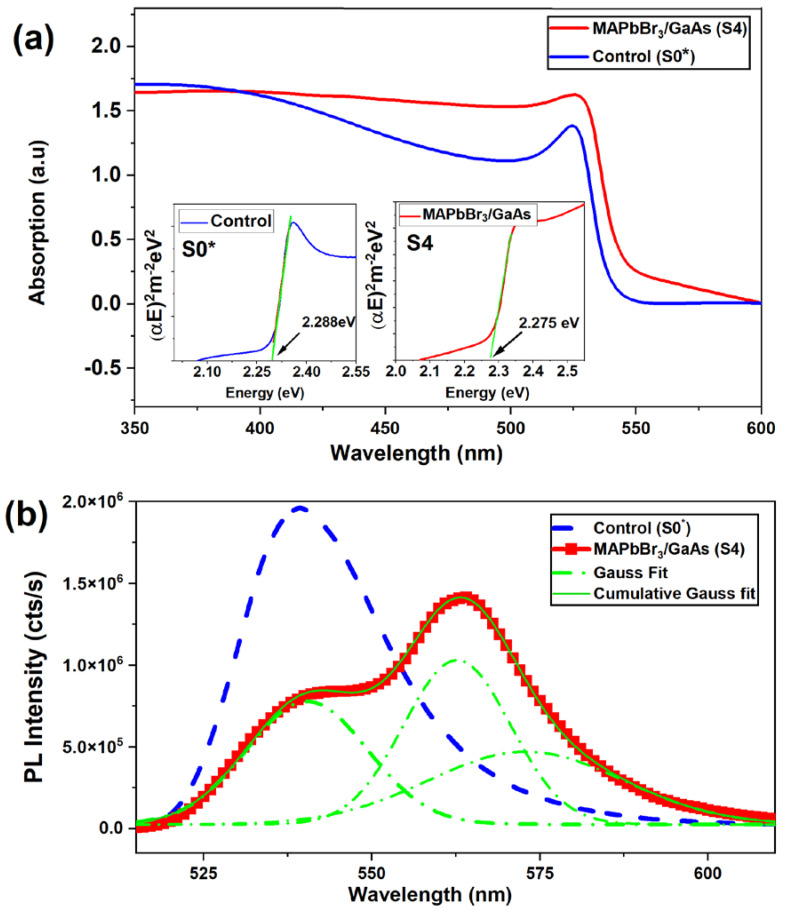

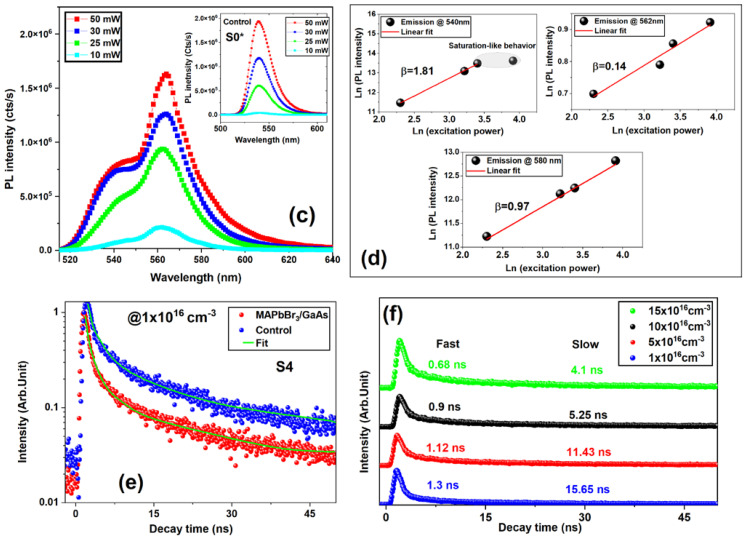
(**a**) Absorption spectra; (**b**) PL spectra taken at 300 K, showing that the broad MAPbBr_3_/GaAs emission is deconvoluted using the Gaussian function (dashed green lines); (**c**) power-dependent PL spectra of MAPbBr_3_/GaAs; inset shows the power-dependent PL spectra of the control (S0 *); (**d**) integrated PL intensity vs. excitation power (black dots) fitted with power law (red solid line); (**e**) time-resolved PL spectra of the control (S0 *) and MAPbBr_3_/GaAs (S4), showing the experimental time trace (dots) with bi-exponential fit (solid lines) at a fixed density of excited carriers; (**f**) time-resolved PL spectra of MAPbBr_3_/GaAs (sample S4) acquired at different densities of excited carriers (different photon density) and the corresponding time constants, using bi-exponential fit.

The absorption spectra of the MAPbBr_3_/GaAs heterostructure are shown in Figure 6a. It clearly shows that the light absorption in the visible region is enhanced with respect to the control sample. This confirms the validity of the designed MAPbBr_3_/GaAs heterostructure for visible light absorption. Due to the difference in structure and thickness (MAPbBr_3_-1.8 µm/GaAs and MAPbBr_3_-867 nm/glass), the intensity of absorption is for reference only. The scattering of light in the hybrid system could improve absorption in the visible region. The excess of MABr may also play a positive role by passivating grain boundaries and minimizing the density of V_Br_-related trap states in the upper layer. Consequently, it will enhance charge carrier movement and decrease the recombination rate.

The Tauc plot in Figure 6a shows a red shift of the bandgap from 2.288 eV (542 nm) in the control sample to 2.275 eV (545 nm) in MAPbBr_3_/GaAs, supported by the change in the absorption onset for MAPbBr_3_/GaAs. The UV-vis spectra of both samples are characterized by several absorption bands (more pronounced for S0 *). For the control sample, this could be ascribed to the presence of bromoplumbate species [36,37]. In the case of deposition on GaAs, such species are mostly prevented, owing to the reaction of bromine with surface arsenic to form AsBr_3_.

Steady-state PL measurements at room temperature are reported in Figure 6b. For both samples, the excitation power density was carefully chosen to avoid surface damaging due to the local heating effect. The control sample shows a clear asymmetric emission that can be fitted using two Gaussian functions. It was found that the most intense PL peak was at 542 nm, with a full width at half maximum (FWHM) of ~18 nm, due to the free exciton emission [38]. The lower energy peak at 562 nm, with a broad FWHM of 32 nm, could be attributed to the recombination through trap states, probably Br vacancies (V_Br_) [38].

Upon integrating the two materials, the heterostructure shows an emission in the visible range (Figure 6b) and low emission from GaAs in the NIR region (Figure S4, Supporting Information). Figure 6b shows that the PL spectrum of MAPbBr_3_/GaAs is characterized by a broad band from 516 nm to 620 nm, with a major flex, which is not present in the reference sample. Such PL quenching indicates that a charge transfer and exciton dissociation at the interface occurred in the MAPbBr_3_/GaAs sample but not in the reference.

A clear shift can be assigned to the change in the grain size and to strain effects connected with the locally variable precursor ratio (revealed by EDS). The main peak can be fitted by three Gaussian functions centered at about 542 nm (2.296 eV), 562 nm (2.206 eV), and 577 nm (2.15 eV). The peak at 542 nm originates from the free exciton emissions of the perovskite layer. The 562 nm and the 577 nm emissions can be associated with defect-related recombination [4,39]. More precisely, we propose that such emissions originate from localized states induced by local potential fluctuations due to the vacancies-induced disorder inside the material [4,5,6]. Disorder can give rise to families of defect states in the bandgap, spatially concentrated in some specific points where carriers are trapped. There might be multiple causes for disorder. First, local fluctuation in stoichiometry (demonstrated by EDX measurements) can lead to local fluctuations in lattice mismatches and residual strain. Second, the bimodal distribution of grains and microcubes can also give rise to energy states and carrier localization. Third, variable V_Br_ concentration can produce higher/lower density of states in the bandgap. Local disorder, both micrometric and sub-micrometric, actually causes emissions assisted by deep states.

To investigate the recombination mechanisms, power-dependent PL was carried out. Figure 6c,d show nearly identical line shapes throughout the entire excitation range. Equation (1) illustrates the excellent power law dependence of the PL intensity on the excitation power.

(1) $$I_{PL}\alpha P^{\beta }$$

where β = 0.14 and 0.97 are both less than the unity, respectively, for the peaks at 562 nm and 580 nm. This suggests that the emission is mostly due to defect-related recombination. For the peak at 540 nm, 1 < β = 1.81 < 2, suggesting an excitonic-like transition, which confirms the excitonic characteristics of spontaneous emission in the proposed heterostructure. The trend for saturation at the high excitation power observed for the 540 nm peak is a signature for defect localization.

Time-resolved PL in Figure 6e shows a reduced profile, indicating a faster PL decay channel for the heterojunction device, and provides direct evidence of electron transfer behavior. It exhibits two decay channels; the first is a fast initial one with a shorter lifetime of τ_1_ = 1.3 ns (~1.95 ns for the control), attributed to the loss of carriers at the interface due to charge extraction or interface recombination. The slow decay channel, with a longer lifetime, τ_2_ = 15.65 ns (22 ns for the control), corresponds to the radiative recombination of free carriers generated far away from the MAPbBr_3_-GaAs interface. From Equation (2), one can estimate the average lifetime. The time constant is slightly larger than that reported for similar heterojunctions, such as CsPbBr_3_/GaN [15], indicating a lower density of defects. The longer carrier lifetime indicates that the nonradiative loss of the excitons was reduced [15].

(2) $$\tau_{avg}=\frac{A_{1}\tau_{1}+A_{2}\tau_{2}}{A_{1}+A_{2}}$$

where $A_{1}$ and $A_{2}$ are constants. $\tau_{1}$ and $\tau_{2}$ are decay times. From Figure 6e, the average lifetime of carriers in sample S4 (13.8 ns) is seen to be reduced compared to the sample S0 * (19.06 ns), which indicates the presence of traps. The short decay time for the MAPbBr_3_/GaAs heterojunction suggests that trapping phenomena are reduced with respect to the control sample. Such a result confirms a faster response speed for the planned detector.

Excitation density dependence is reported in Figure 6f. By increasing the excitation density, the long lifetime decreases from 15.65 ns to 4.1 ns, and the short lifetime reduces from 1.3 ns to 0.68 ns. Both decay channels follow a trend of the type τ ∝ 1/n, where n is the excitation density [40]. The appearance of an interface recombination/extraction decay channel suggests a sharp interface between MAPbBr_3_ and GaAs, which would provide a good platform to develop hybrid functional optoelectronic devices. Furthermore, the change in the larger decay time can also be correlated with the morphology of the film. Indeed, an excess of MABr is thus important not only for improving the coverage but also for reducing the nonradiative losses.

**Figure 7:**
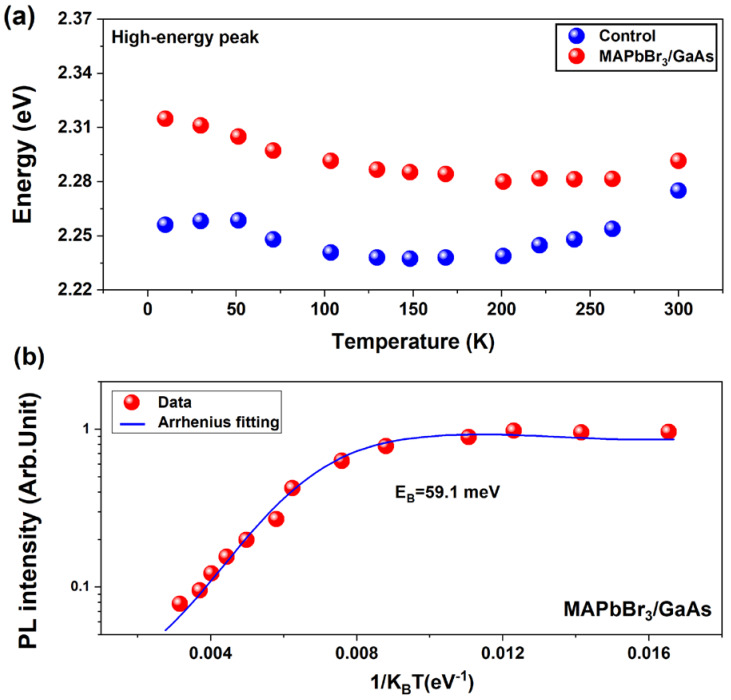
(**a**) Temperature dependence of the PL emission energy of the high-energy peak of MAPbBr_3_/GaAs (S4) (red dots) and its control sample (blue dots). (**b**) Temperature-dependent PL intensity of MAPbBr_3_/GaAs (red dots) fitted by Arrhenius law.

Temperature-dependent PL for both samples is reported in Figure 7a. The crystallographic phase of MAPbBr_3_ bulk crystals has been reported to be cubic, tetragonal, and orthorhombic, at room temperature, at ~250 K, and at ~150 K, respectively. The control sample shows a redshift of the optical bandgap as the temperature decreases and passes from 2.275 eV to 2.235 eV around 150 K, corresponding to a crystal phase change from tetragonal to orthorhombic. Then, it shows a blueshift up to 2.26 eV at 50 K. Finally, the PL peak moves slightly again to the lower energy side for temperatures lower than 50 K. The main energy peak of the control shows a blueshift upon increasing temperature, except for the range around 150–160 K due to the orthorhombic-to-tetragonal phase transition. This blueshift has previously been assigned to the expansion of the lattice and stabilization of the valence band maxima [41]. In the MAPbBr_3_/GaAs heterostructure, the variation in PL emission with temperatures between RT and 50 K is milder, without an evident formation of the orthorhombic intermediate phase. This may be due to the excess in MABr, which tends to minimize the formation of that phase, together with the high reactivity of the MAPbBr_3_ on the GaAs surface following the preparation method. The apparent absence of the orthorhombic phase is important, as it makes the perovskite structures stable over a wider operating temperature range below RT. In both samples (MAPbBr_3_/GaAs and the control), importantly, the low energy peaks at about 565 nm display a similar trend as a function of temperature. This means that the two samples contain the same type of defect trap. Moreover, the low energy peak shows a less pronounced two-step redshift transition (around 150 K), meaning higher stability of the sample. The pronounced redshift as the temperature increases can be explained in terms of electron–phonon coupling [42]. However, in this work, we focus on the high-energy peak that shows a different trend in the MAPbBr_3_/GaAs heterostructure. It exhibits a blueshift at temperatures above ~220 K, near the cubic–tetragonal phase transition. In the tetragonal phase region, the peak redshifts with increasing temperature. This redshift cannot be attributed to the enhanced electron–phonon coupling, as the FWHM of the investigated PL peak is narrower than that of the pristine MAPbBr_3_. It has been suggested that a slight redshift in the bandgap due to electron–phonon coupling is typically observed in small nanocrystals but not in bulk perovskites. Instead, the actual temperature dependence of the high-energy PL peak in MAPbBr_3_/GaAs suggests that reduced orthorhombic tilting, also leading to redshift, plays a more significant role than the thermal expansion of the lattice. Furthermore, the MABr excess introduces shallow level defects that, along with band-to-band recombination, results in a double-peaked feature, as evidenced by the Gaussian fit. That could redshift the emission. Exciton binding energy (E_B_) shows the interaction strength of the electrons and holes and can be extracted by fitting the PL emission data using Equation (3).

(3) IT=I01+Ae−EBKBT

where I_0_ is the PL intensity at 0 K, A is a fitting parameter, and K_B_ is the Boltzmann constant. The calculated exciton binding energies of the control sample and MAPbBr_3_/GaAs are 72.4 meV and 59.1 meV, respectively. The low exciton binding energy in the investigated heterostructure indicates an easier separation of generated carriers during the device operation process. This is beneficial to enhance the figure of merit of the photodetector and the charge transfer between both layers.

### 3.2. Structural and Photo-Stability

**Figure 8:**
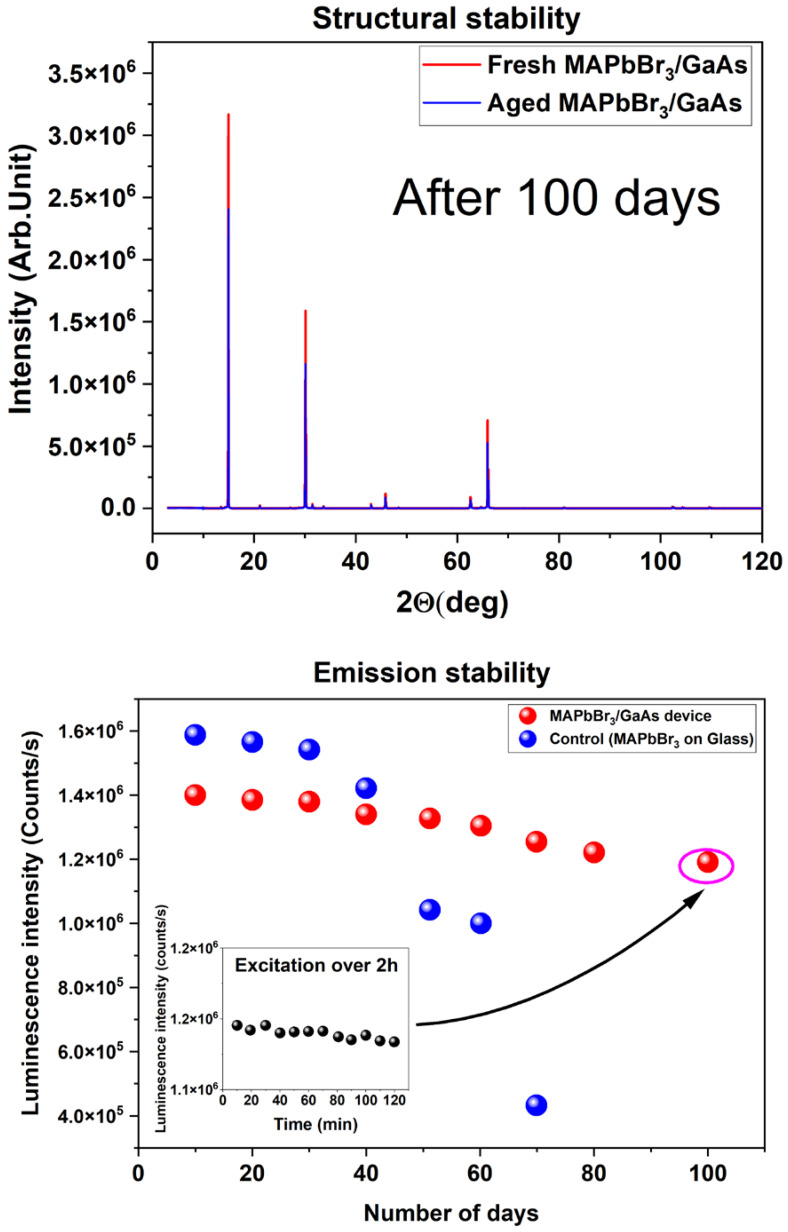

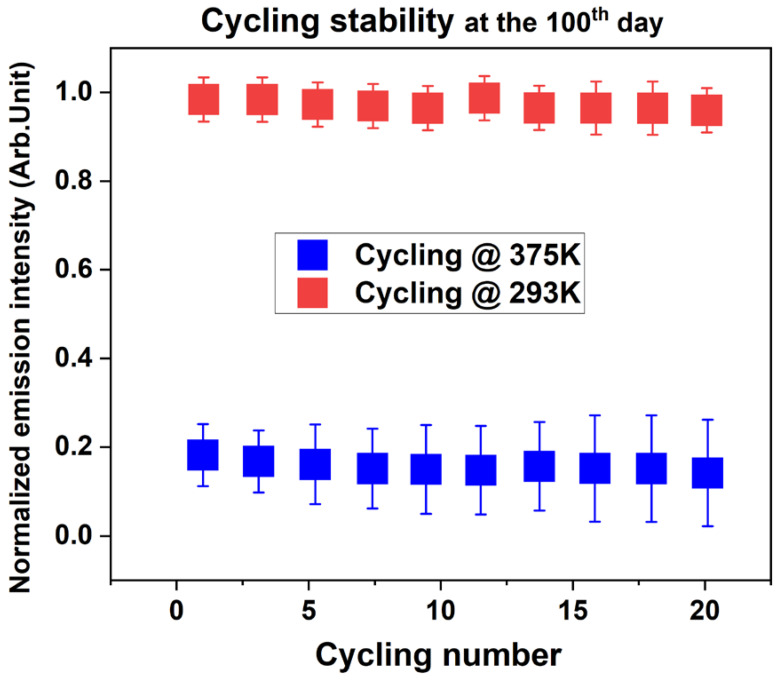
Structural, optical, and cycling stability of the MAPbBr_3_/GaAs heterojunction (sample S4).

Measurements of structural stability, long-term emissions, and cycling of the heterostructure and control sample in ambient conditions, without any encapsulation and protection, were also carried out. The results are compiled in Figure 8. Both samples were exposed to laser light directly at room temperature for two hours per day, and intensity was measured at the end of the second hour.

Both structures were tested again at 100 days (Figure 8). From a structural point of view, it must be noted that both samples show no additional peaks with respect to those freshly prepared. In particular, there is no peak at 2θ = 9.6°, which corresponds to the (001) plane of MABr. This allows us to conclude that there is no apparent decomposition of the MAPbBr_3_ film. The developed preparation and deposition method thus seems to be effective against degradation in an unprotected ambient. The hybrid heterostructure preserved its emission properties almost for the entire period, whereas the control sample was considerably less stable and started the degradation earlier. To investigate the stability of the structure over the illumination period, the sample was excited continuously, and the PL intensity was recorded every 10 min in the last day of storage (after 100 days). The structure was seen to be stable under continuous light illumination over two hours, with a good PL recovery over twenty cycles of heating–cooling. Such good stability is associated with the stable substrate, the low sensitivity of Br to the moisture, and the reduced ion migration resulting from the expanded grain size. The enhanced stability is mainly due to the nonstoichiometric preparation conditions that slow down the crystallization rate. The above results confirm the improved material stability of the hybrid structure, which is an important factor for application, as photodetectors operate in harsh environments.

### 3.3. Photoelectrical Measurements

**Figure 9:**
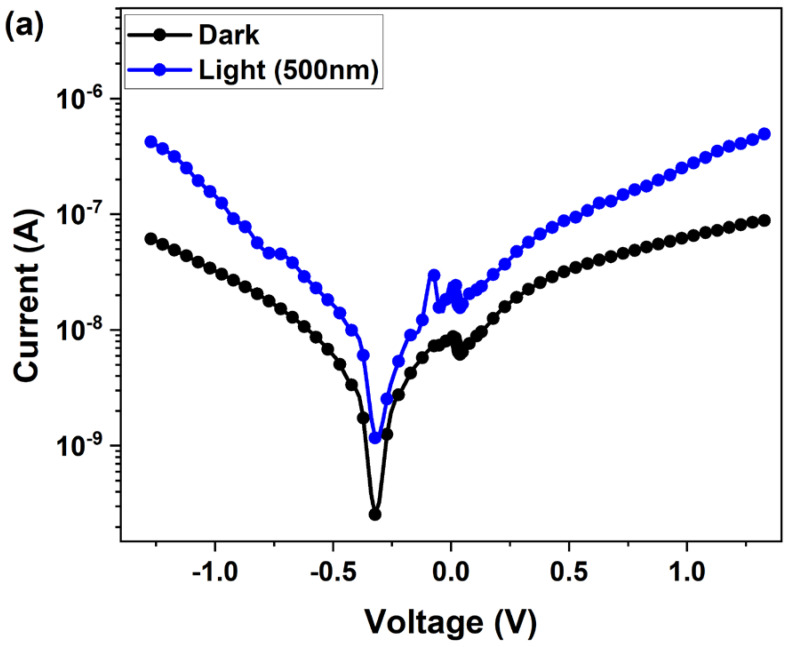

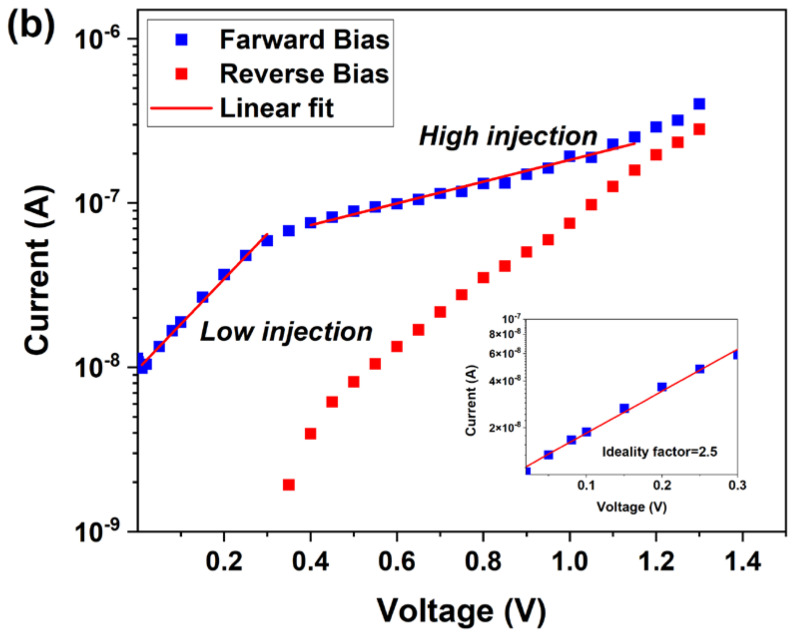
(**a**) I–V response in semi-log scale in the dark (black symbols) and under 500 nm light illumination (blue symbols). (**b**) Room-temperature dark forward current–voltage characteristics in a semi-log scale. The linear fitting (red solid line) of ln(I)–V is shown. Inset: the obtained ideality factor extracted from the low-injection region.

Figure 9a reports the current–voltage (I–V) characteristics of the heterojunction photodetector in the dark and under illumination with 500 nm light and an applied bias of ±1.25 V. Under illumination, an enhancement of the current was observed over the whole bias range (±1.25 V). The shape of the I-V curves is similar to that reported in Ref. [6]. The weak rectification effect is similar to that observed in SnO_2_/CsPbBr_3_ and Ga_2_O_3_/Au/MAPbBr_3_ heterojunctions [6,13] and is mainly due to the undoped MAPbBr_3_.

Figure 9b shows the fitting of the linear region of the forward bias ln(I)–V characteristics and the extracted ideality factor from the low-injection region (low forward bias) based on the thermionic emission theory. The measured value is 2.5, higher than the unity, indicating the presence of impurities, interface states, and recombination in the depletion region. The recombination is probably assisted by in-gap centers and interfacial states that dominate the carrier transport process at the MAPbBr_3_/GaAs interface. The high-injection region exhibits a linear trend consistent with a tunneling transport across the perovskite/metal interface, indicating a Schottky-like barrier of about 0.35 V. The measured dark current is ~8.8 × 10^−8^ A at 1.2 V, while the maximum measured photocurrent is 5.4 × 10^−7^ A. Ion migration is one of the considerable factors that limit the increase in current.

Strangely, the I–V curves show an offset of about 0.3 V towards the negative side of the axis. This character is observed in other photodiodes, and it may be due to the hysteresis cycle, as well as cation accumulation in the interface or between the metal contact and the perovskite layer.

**Figure 10:**
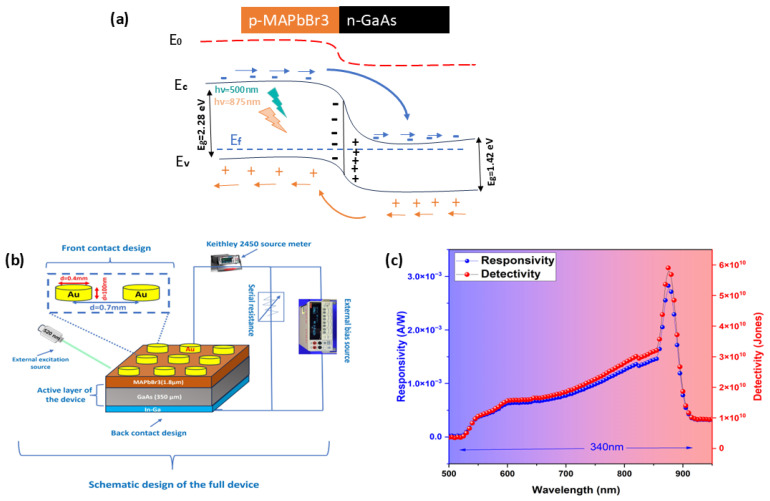
(**a**) Schematic energy band diagrams of the junction (S4) under illumination and reverse bias. (**b**) Schematic design of the final device and (**c**) the extracted responsivity and detectivity under 500 nm illumination and bias of −1 V.

To the best of our knowledge, this is the first experimental report on a photodetector prototype based on the MAPbBr_3_/GaAs heterojunction. With respect to vacuum level, the bottom of the conduction band (CB) of GaAs and MAPbBr_3_ is located at −3.98 eV and at −3.38 eV, respectively, while the top of their valence bands (VBs) is located at −5.4 eV and at −5.68 eV, respectively. Considering the fact that the MAPbBr_3_ films exhibit weak p-type conductivity [2,5] with a hole concentration between 5 × 10^9^ and 5 × 10^10^ cm^−3^, and that the GaAs is weakly doped silicon with Si (n ~ 3–4 × 10^17^ cm^−3^), a p–n heterojunction can be established once the MAPbBr_3_ films and GaAs are in contact. Thus, a built-in electric field forms at the hetero-interface due to the realignment of the Fermi levels in the two materials. Upon illumination using 500 nm source (Figure 10a), electron and hole photocarriers will be mostly generated in the depletion region of the perovskite layer and then separated by the electric field created by the built-in and the external bias. Electrons will drift from the CB of the MAPbBr_3_ to the GaAs side and will be further collected by the metal cathode. At the same time, holes will move in the opposite direction and will be transported into the VB of MAPbBr_3_, then collected by the upper anode. This photovoltaic process generates the photocurrent in the external circuit, while keeping a low reverse dark current and enhanced detection capability. A schematic drawing of the heterojunction photodetector is shown in Figure 10b.

The spectral photoresponse (Figure 10c) of the MAPbBr_3_/GaAs heterojunction demonstrates the responsivity of ~3 mA/W. The cutoff wavelength was around 875 nm, which corresponded to the GaAs edge of the energy band. Illumination using 875 nm at room temperature (Figure 10c) actually produces a peak of responsivity due to the light absorption in the GaAs bandgap. Light with such wavelengths penetrates the MAPbBr_3_ thickness and reaches the GaAs substrates. In this case, both the surface-generated carriers and bulk-generated carriers can be collected by electrodes, resulting in the broadband spectral response. Because of the low thickness of the perovskite (1.8 μm or less), the stronger distribution of the electric field intensity in the GaAs, and its higher quantum efficiency in the NIR region, the GaAs layer has higher responsivity than the MAPbBr_3_ layer at 875 nm. Such a characteristic is confirmed by measuring the responsivity at low voltage (−0.5 V).

**Figure 11:**
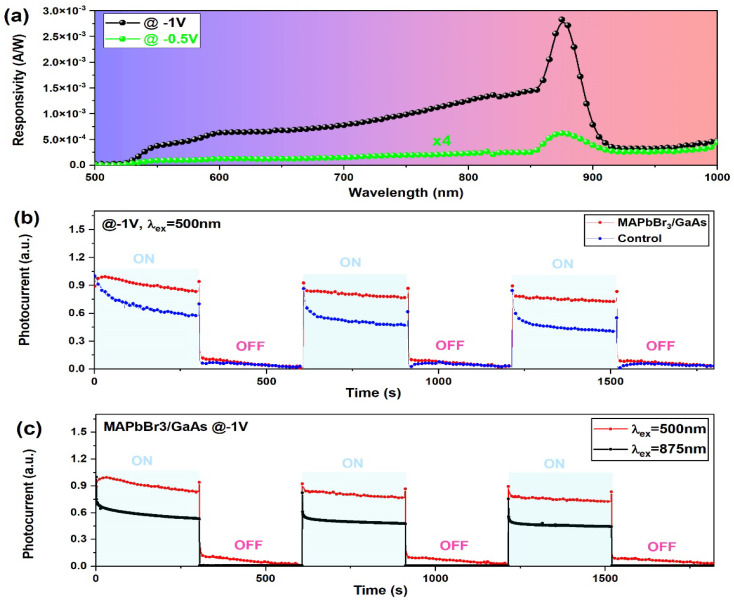
(**a**) Responsivity of MAPbBr_3_/GaAs under −0.5 V (green symbols) and −1 V (black symbols) for illumination with 500 nm photons. (**b**) Time-dependent photoresponse showing the on/off switching cycles for 500 nm illumination at −1 V of the sample S4 (red dots) and the control sample S * (blue dots). (**c**) Time-dependent photoresponse of MAPbBr_3_/GaAs under 500 nm and 875 nm illumination at a constant bias (−1 V).

Figure 11a shows that the device is highly sensitive to external voltage. Indeed, changing the bias from −0.5 V to −1 V increases the responsivity by ~19 times. This is due to the more effective separation of the photogenerated electrons and holes and the effect of the GaAs substrate. Indeed, for higher biasing, the external quantum efficiency will increase monotonically. This high efficiency will maintain higher responsivity, even under higher biasing of 50 V. The responsivity indeed can reach 16 mA/W (Figure S5, Supporting Information), indicating the appropriate carrier drifting length and depth of the junction.

The ability to distinguish a signal from noise (signal-to-noise ratio when one watt of light power is incident on a detector of an area of 1 cm^2^) or the detection capability of the photodetector to monitor weak signals is defined as special spectral detectivity D*. It is directly related to spectral responsivity R and the intrinsic noise of the detector. The noise equivalent power (NEP) represents the minimum optical signal power that can be resolved by the photodetector and is related to the other entities according to the following set of relations:

(4) $$R=\frac{I_{ph}-I_{d}}{P_{ex}S}$$

(5) $$NEP=\frac{R\sqrt{S}}{D^{*}}$$

(6) D*=RS2eId

where S is the effective area of the photodetector, *e* is the electron charge, I_ph_ is the photocurrent, and *I*_d_ is the dark current. The maximum detectivity of 6 × 10^10^ Jones (cm Hz^1/2^ W^−1^) is achieved at 875 nm (−1 V), showing a broadband photodetection characteristic ranging from the VIS to NIR regions. The smallest optical signal power, NEP, that can be resolved from the photodetector noise is calculated from the special detectivity to be NEP = 376 × 10^−18^ W/Hz^1/2^. The time-dependent photoresponse (Figure 11b,c) was determined under illumination at 500 nm. The MAPbBr_3_/GaAs-Au heterojunction-based photodetector exhibits a good cycling response, and the “ON/OFF” switching shows good stability and reproducibility.

The response time is defined as the time taken to rise from 10% to 90% (rise time) or from 90% to 10% (decay time) of the current signal. Figure 11b shows the transient response measurement of the device under 500 nm laser excitation at room temperature. The power is fixed at 40 μW under a biasing of −1 V. It should be noted that there is a reproducible peak when the light turns on. This is attributed to a pyroelectric effect caused by the sudden temperature change in the detector upon illuminating the device. The device then exhibits a relatively stable response until switching off. The behavior towards periodic intermittent light is very reproducible, which is an important parameter for practical application. By fitting a single normalized cycle, the rise time (*τ*_r_) and the fall time (*τ*_f_) constants are 0.4 s and 0.61 s, respectively. This is shorter than values reported in similar devices such as MAPbBr_3_/Si and MAPbI_3_/GaN detectors [14,20]. The same switching cycles for the control sample give values of 0.77 s and 0.84 s for the rise and decay time, respectively. The faster rise response of the heterostructure was attributed to the larger grain size and to the more effective separation of photogenerated carriers, thanks to the additional built-in electric field at the interface. This validates the proposed recipe that combines the change in soaking time and stoichiometric conditions. The decay time strongly depends on trap states at the interface of the heterojunction. Thus, it seems that the MAPbBr_3_/GaAs system (S4) introduces fewer defects and fewer related traps.

To show the validity of the proposed recipe in terms of stoichiometry, switching cycles under 875 nm illumination (Figure 11c) were applied for the sample S *. Even at stoichiometric conditions and with a lower generated photocurrent, the proposed structure can respond to light excitation, showing rise and fall times of 0.62 s and 0.89 s, respectively. The results confirm the ability of the proposed junction to work in a dual-wavelength mode. Note that the apparent lower photocurrent at 875 nm, with respect to 500 nm, is only due to normalization and to the different trends in time when the light turns on (considering the peak generated by the pyroelectric effect that appear upon a sudden local increase in temperature in on/off cycling). Additionally, the initial sharp peak at 875 nm does not correspond to a sharp peak at 500 nm. As a consequence, the photocurrent at 875 nm is more reduced after normalization.

A comparison of the present heterojunction device with other photodetectors is shown in Table 2. The responsivity is generally superior, but the response speed of the present device is relatively slow compared with some organic–inorganic hybrid perovskite photodetectors based on asymmetric electrodes, probably because the carrier diffusion time is shorter in Schottky-based devices with asymmetric electrodes than in p–n junction devices.

We think that the device’s performance can further be enhanced. Firstly, it can be enhanced by optimization of the electrode contact on MAPbBr_3_ film to minimize the undesired interface states at the MAPbBr_3_/Au interface, where a number of charge traps are still present. Secondly, it can be enhanced by improving the size uniformity and spatial arrangement of the crystallites within the MAPbBr_3_ film. These tiny crystals are arranged in an arbitrary manner, which complicates the transfer of photocarriers to the electrodes, in addition to promoting recombination on their surfaces.

## 4. Conclusions

In summary, films of MAPbBr_3_ perovskite were successfully deposited on GaAs, with improved coverage and adhesion. The established deposition method was based on a modified multi-step spin coating method and an appropriate antisolvent soaking time. HR-XRD and SEM showed that MAPbBr_3_ film directly deposited on GaAs are made of large grains separated by grain boundaries. They exhibit better crystallinity than in stoichiometric specimens on glass or deposited on GaAs with no antisolvent soaking, with a surface made of small cubic particles floating on the continuous underlying film. The deposited films showed a uniform distribution of chemical elements on the macroscale, but on the microscale, local composition fluctuations were confirmed by SEM-EDX. Grain size inhomogeneity may cause different concentrations of vacancies in the bulk of the cubes owing to the tendency for Br and MA to accumulate towards the cube surfaces (more or less extended) and leave corresponding vacancies behind. Optical measurements showed that the absorption of MAPbBr_3_/GaAs was enhanced in the visible range and redshifted by 13 meV, while the PL emission was broadened and quenched. This emission is governed by free excitons and carrier localization ascribed to the effects of bromine vacancies, local composition, and strain fluctuations, as suggested by the power-dependent PL measurements. Temperature-dependent PL showed a reduction in the orthorhombic phase, which enhanced the long-term optical and structural stability of the fabricated structure. The TRPL showed a reduction in the intensity average lifetime from 19.06 ns in the sample on glass to 13.8 ns for MAPbBr_3_ on GaAs, which was probably connected with faster photocarrier recombination and lower trapping in the improved perovskite deposited on GaAs.

VIS-NIR MAPbBr_3_/GaAs photodetectors prepared under stoichiometric and non-stoichiometric conditions, keeping the same soaking time, generated a photocurrent of 5.4 × 10^−7^ A and exhibited a rise/fall time ratio of 0.4 s/0.61 s and 0.62/0.89 s under on/off light switching at 500 nm and 875 nm, respectively, which makes them suitable for dual-wavelength detection. The broaden responsivity over 330 nm reached a maximum of 3 mA/W (16 mA/W at 50 V), while the detectivity was 6 × 10^10^ Jones at −1 V (1 × 10^11^ Jones at 50 V), and the noise equivalent power was 376 × 10^−18^ W/Hz^1/2^. The overall device performance was comparable to or better than that reported for most hybrid heterostructure photodetectors.

These results may open the way to prepare more efficient APbX_3_/GaAs-based photodetectors or luminescent/absorbent hybrid heterostructures. To this extent, new additives must be found to suppress the nonradiative recombination, and carrier transport layers may be inserted in the structures. These present findings can thus help the development of electronic and optoelectronic devices working at room temperature, as well as at low temperatures.

## Figures and Tables

**Table 1:** Film preparation conditions for the spinning of MAPbBr_3_ on both GaAs and reference glass substrates, including stoichiometry and soaking procedures (0.5 mL of anti-solvent added in one or more shots).

	Stoichiometry (MABr:PbBr_2_)	Soaking Time and Procedure	Sample Symbol and Role
MAPbBr_3_/GaAs	(1:1)	10 s (single shot)	S *, control
MAPbBr_3_/glass	(1:1)	10 s (single shot)	S ^g^
MAPbBr_3_/glass	(3:1)	10 s (single shot)	S0, control
MAPbBr_3_/glass	(3:1)	15 s + (4 × 5 s) = 35 s (multiple shots)	S0 *, control
MAPbBr_3_/GaAs	(3:1)	10 s (single shot)	S1
MAPbBr_3_/GaAs	(3:1)	5 s + 5 s = 10 s (multiple shots)	S2
MAPbBr_3_/GaAs	(3:1)	10 s + 5 s = 15 s (multiple shots)	S3
MAPbBr_3_/GaAs	(3:1)	15 s + (4 × 5 s) = 35 s (multiple shots)	S4 (Optimized)

**Table 2:** Comparison of the achieved responsivity of the MAPbBr_3_/GaAs VIS-NIR Photodetector with respect to previously reported photodetectors.

Materials and Structures	Responsivity (mA/W)	Conditions: Voltage/Light Excitation	Preparation Method	Reference
In-Ga/GaAs/MAPbBr_3_/Au	3 16	@ −1 V, 500 nm @ 50 V, 500 nm	Spin coating	This work
Au/MAPbBr_3_ SC/Au Plane MSM-photodetector	1.7	@5 V, 532 nm	Unchanged temperature	[18]
Pt/MAPb(Br*_x_*I_1−*x*_)_3_ SC/Pt	2.41	@ 10 V, white light	Inverse temperature crystallization (ITC)	[2]
Au/MAPbI_3_ nanosheets/SiO_2_/Au	0.5	@ 1 V, 635 nm	Chemical vapor deposition	[14]
Cs_3_Cu_2_I_5_/β-Ga_2_O_3_	2.3 3	@ 0 V, @ −3 V, 265 nm	Dual-source vapor co-deposition	[19]
MAPbBr_3_/Si/In	0.394	@ −1 V, 532 nm	Spin coating	[14]
Si/Ga_2_O_3_/MAPbI_3_	1.6	@ 1 V, 780 nm	PLD + Spin coating	[21]
MAPbI_3_/GaN	110 × 10^−6^	@ 0 V, 405 nm	Two-step spin coating	[20]
*β*-Ga_2_O_3_/Au/MAPbBr_3_	1.63 1.40	@ 0 V, 240 nm @ 0 V 520 nm	MBE + spin coating	[6]
SnO_2_ MWs/CsPbBr_3_	347 × 10^−3^	@ 3 V, 530 nm	Vapor transport, drop casting	[13]
ITO-MAPbI_3_-TiO_2_-ITO	0.49 × 10^−6^	@ 3 V, white light	Spin coating	[9]

## Data Availability

Data will be made available on request.

## Supplementary Materials

The following supporting information can be downloaded at: https://www.mdpi.com/article/10.3390/nano14181472/s1.
