# Supplementary material for: Physical Properties of an Efficient MAPbBr3/GaAs Hybrid Heterostructure for Visible/Near-Infrared Detectors
Source: Nanomaterials (Basel). 2024 Sep 10;14(18):1472. doi: 10.3390/nano14181472 (PMC11434396; doi:10.3390/nano14181472)
Supplement: Supplementary file 1 [file nanomaterials-14-01472-s001.zip › nanomaterials-3159077-supplementary.pdf]

Supporting information

Note S1: As mentioned in Table 1, the time of soaking (one shot or multi shots) means dropping the quantity of the anti-solvent over a specific time upon spinning. For example, for 10s one shot, the anti-solvent is dropped once over 10s. If the soaking time is over multiple shots (i.e. 5s + 5s), the anti-solvent can be dropped continuously for 5s, waiting until the color of top layer starts to change (yellow-orange), then one can drop the rest of the quantity over other 5s. As a result, a uniform color must appear.

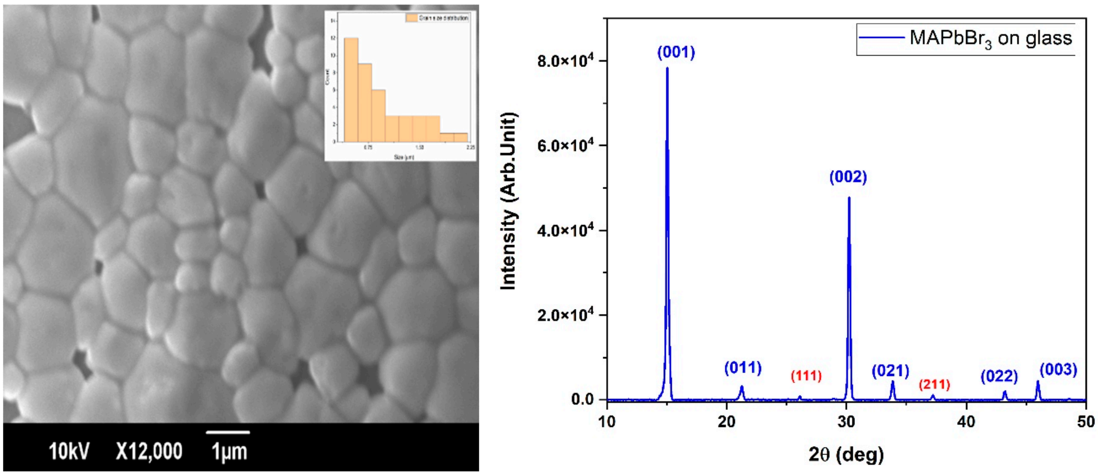

Figure S1: SEM image and XRD profile of $MAPbBr_3$ on glass substrate (sample S0). Inset of the SEM image shows the average grain size.

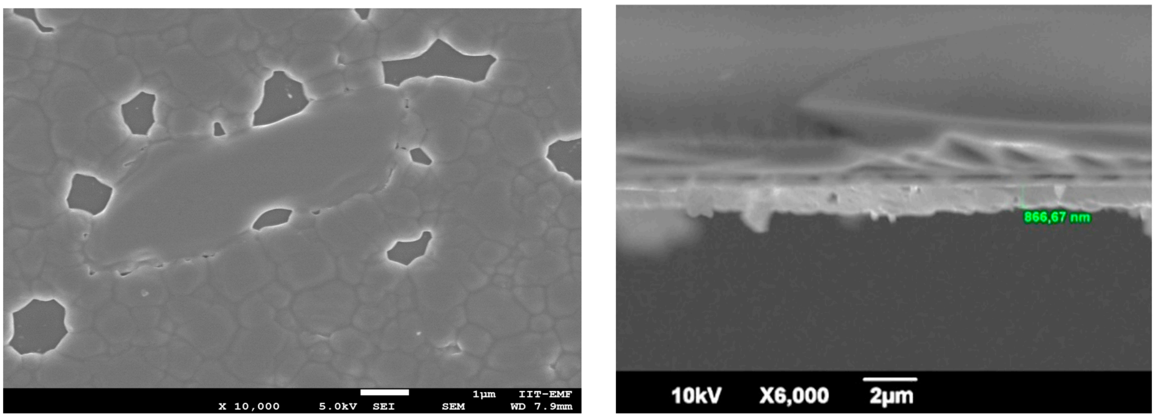

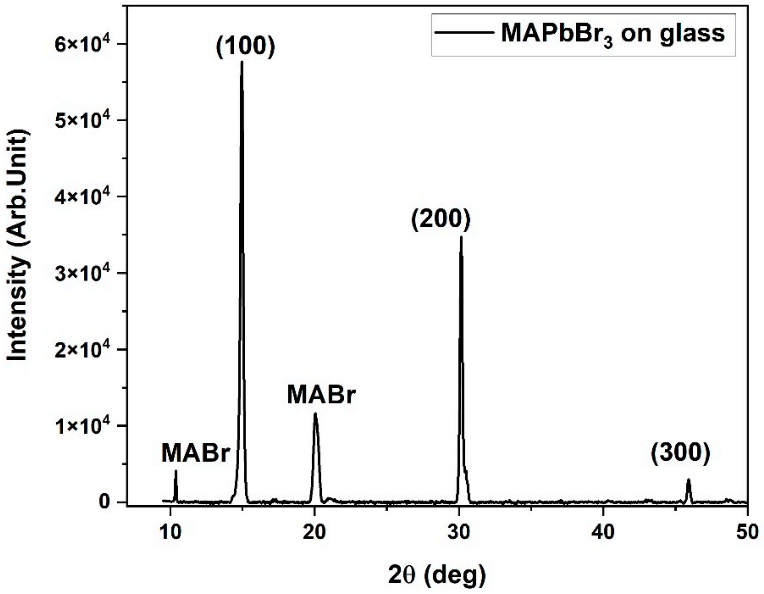

Figure S2: Top-view High Resolution SEM (HR-SEM), Cross-sectional SEM and the XRD diffractogram of the sample S0*.

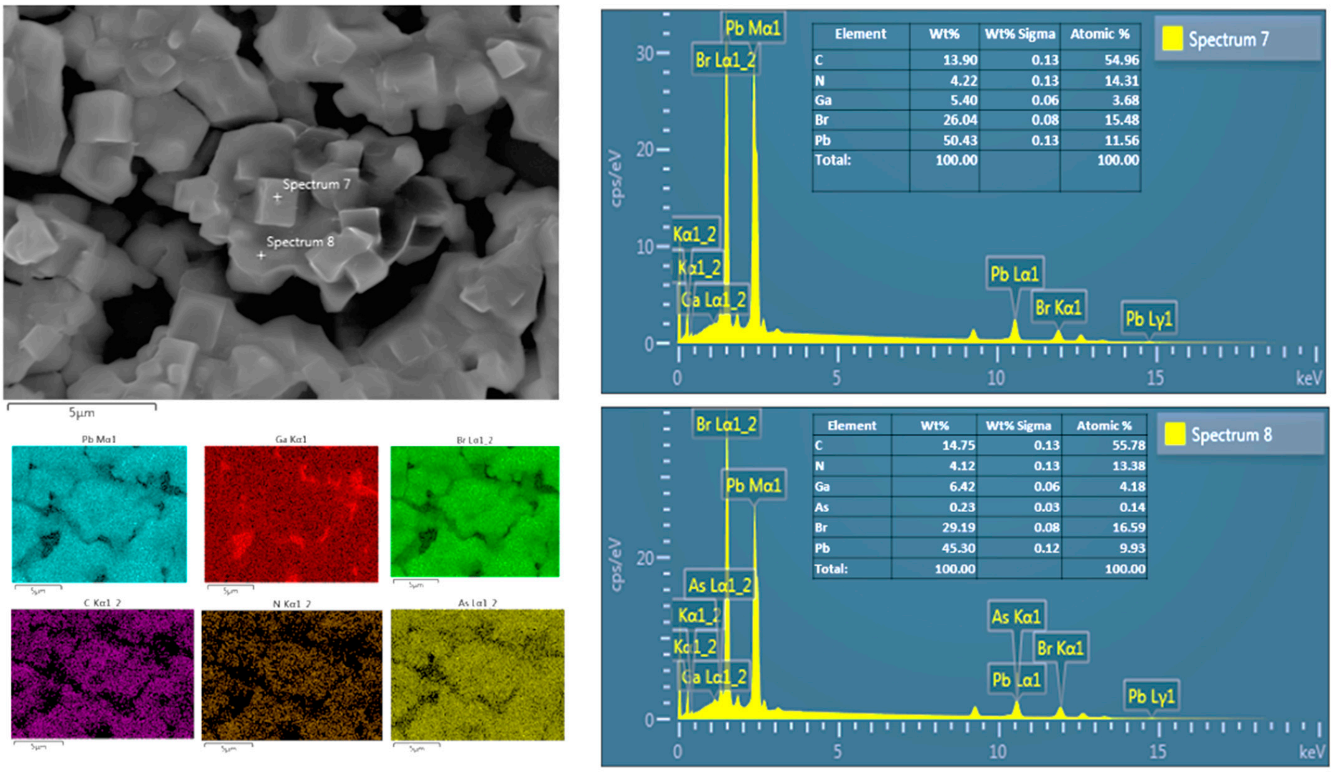

Element	Wt%	Wt% Sigma	Atomic %
C	13.90	0.13	54.96
N	4.22	0.13	14.31
Ga	5.40	0.06	3.68
Br	26.04	0.08	15.48
Pb	50.43	0.13	11.56
Total:	100.00		100.00

Element	Wt%	Wt% Sigma	Atomic %
C	14.75	0.13	55.78
N	4.12	0.13	13.38
Ga	6.42	0.06	4.18
As	0.23	0.03	0.14
Br	29.19	0.08	16.59
Pb	45.30	0.12	9.93
Total:	100.00		100.00

Figure S3: Local EDS mapping and the corresponding local element analysis of $MAPbBr_3$/GaAs taken for the upper layer (spectrum7) and the bottom layer (spectrum8).

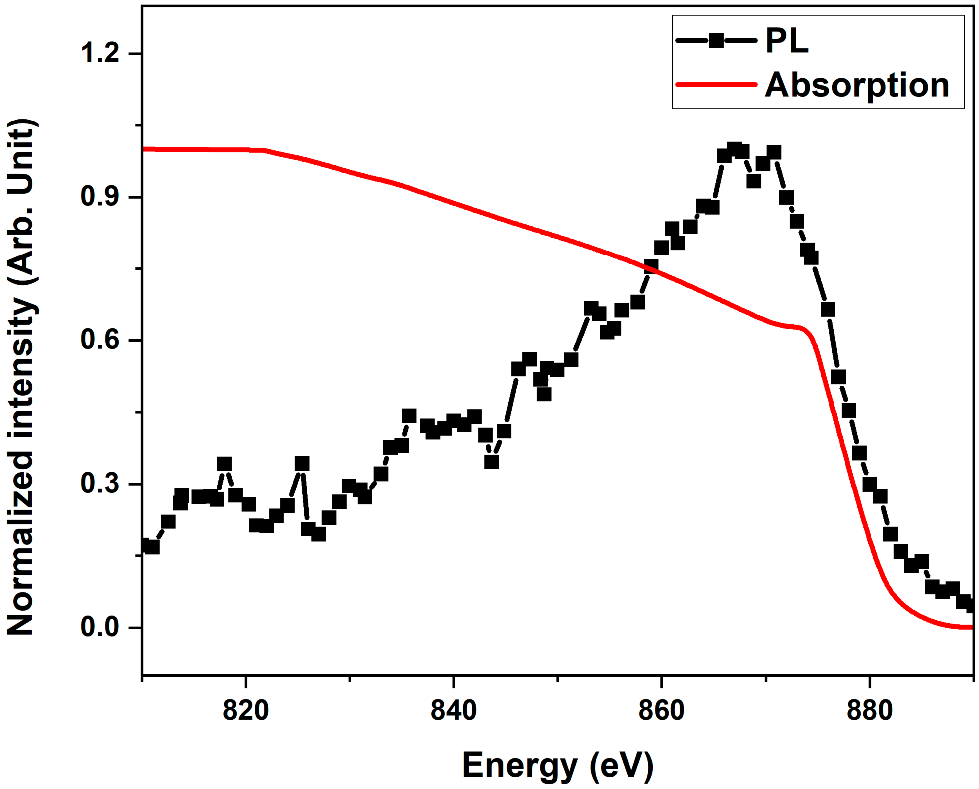

Figure S4: Steady-state PL spectrum acquired using 500 nm excitation (black symbols) and absorption (red solid line) spectra in the NIR region of $MAPbBr_3$/GaAs (sample S4).

Note S2: Discussion of the EDS mapping on the edge

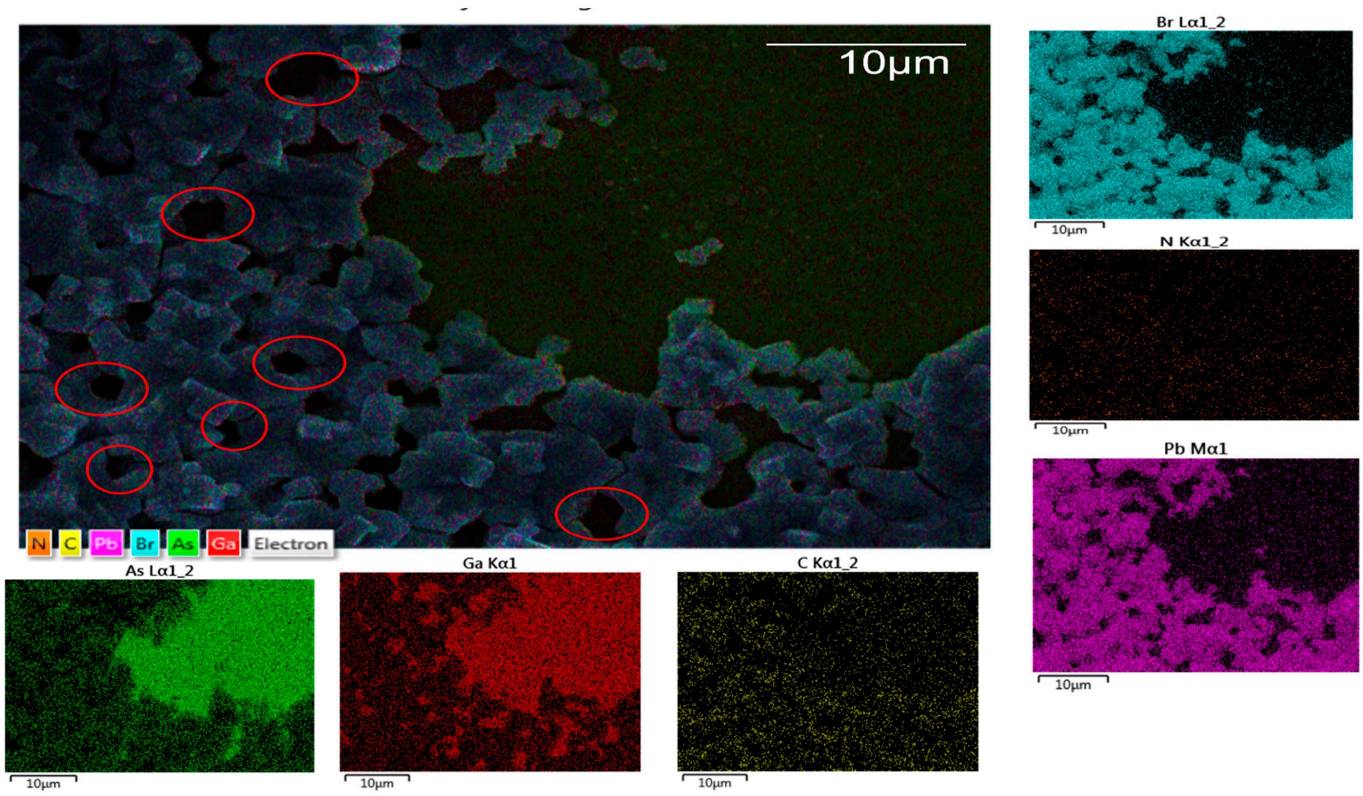

SEM-EDX have been performed on the edge of the sample. It is clear that the inequal diffusion leads to more separated cubes and more pinholes even with equal distribution of perovskite elements. We must note that in such zones, the agglomeration is not total. This may be due to the local strain between the perovskite and the GaAs as well as the arsenic(III) bromide, the arsenic(V), lead- oxides like PbO or PbO_2 that inhibits the total formation of the material which lose a part of the perovskite composition leading to some local composition fluctuations that affect the material properties. Unambiguous identification of these minor compounds is out of the scope of the present work, however, lead oxides, are the most reasonable candidates. The oxide is inhomogeneously distributed over the surface and limits the perfect reactivity between different elements and leads to desorb the precursors which minimize its atomic ratio (see elemental mapping in supporting information). On the edge, all those effects can be well seen which are derived by the limited diffusion of precursor elements near the edge (see supporting information SEM image) which increases the pinhole density (red circle in SEM image of the supporting info).

Instead, except edges, generally an homogeneous distribution of elements on the surface is observed.

Gallium segregation toward the perovskite layer is detected in the upper layer of perovskite. In figure S3, EDS mapping shows both gallium and arsenic diffusion towards the perovskite layer. Such a slight difference in composition maybe due to reactions that can occur between precursors and the diffused gallium and/or arsenic from the GaAs upon heating. The reason behind the absence of the As in the perovskite cubes (figure S3 spectrum7) is that all arsenic is blocked by the perovskite bottom layer (forming $AsBr_3$) and cannot reach the upper single cubes. Such a non-equilibrated diffusion of gallium and arsenic from GaAs is the result of non-equilibrated thermal annealing on the hot plate which leads to the formation of the observed pinholes. This supported by mapping other zones in which the density of pinholes is decreased with a homogeneous distribution of elements. Thus, after the initial nucleation, the crystals grow until they reach each other and merge to form a continuous solid layer. The presence of diffused Ga/As and the GaAs oxidation can inhibit such a mechanism.

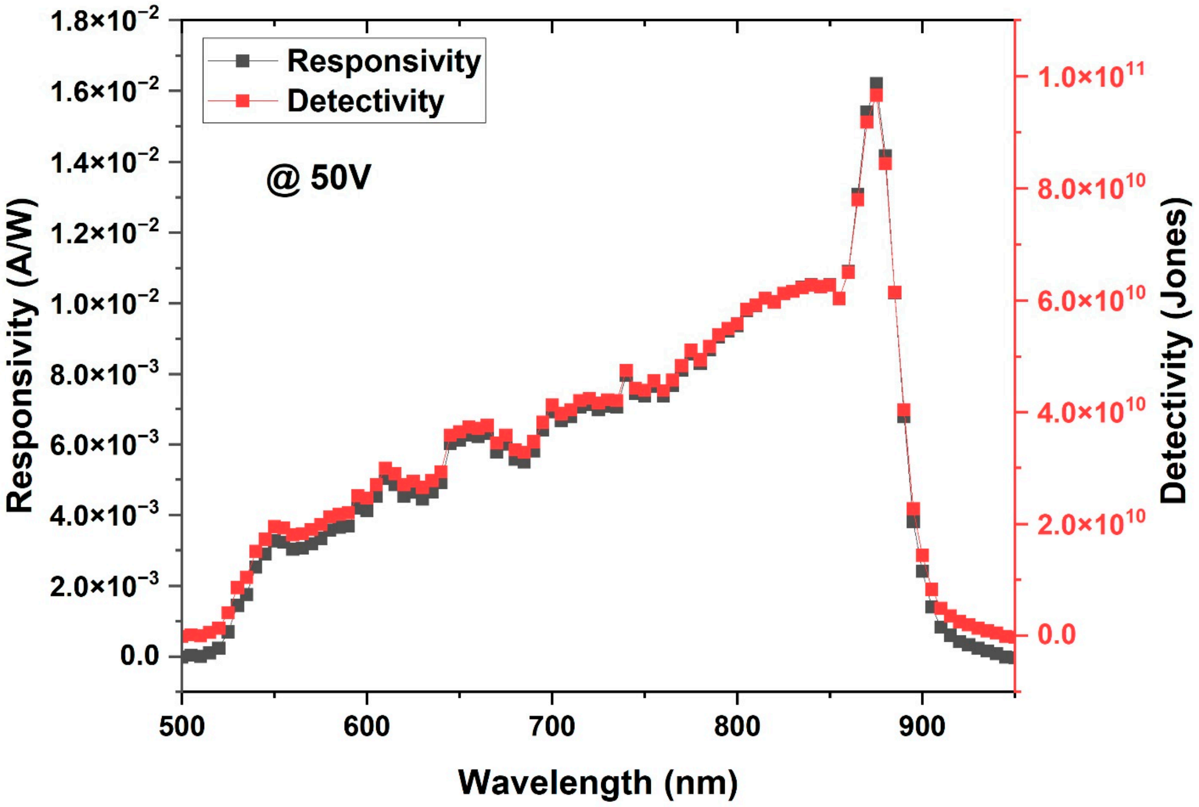

Figure S5: Spectral photoresponse and detectivity under 50 V of $MAPbBr_3$/GaAs (sample S4).
